# Supplementary material for: In vivo investigation of STN1 downregulation in melanoma formation in adult mice following UV irradiation
Source: PLoS One. 2025 Nov 14;20(11):e0326647. doi: 10.1371/journal.pone.0326647 (PMC12617882; doi:10.1371/journal.pone.0326647)
Supplement: S1 Fig — (A) Uncropped gel images for Fig 2B. (B) Original uncropped IHC images. Boxed areas indicate those used for quantification in Fig 4. (PDF) [file pone.0326647.s001.pdf]

Figure legend in Supplemental Files:

A. Uncropped gel images for Figure 2B.
B. Original uncropped IHC images. Boxed areas indicate those used for quantification in Figure 4.

Supplemental. Knowles et al.

A

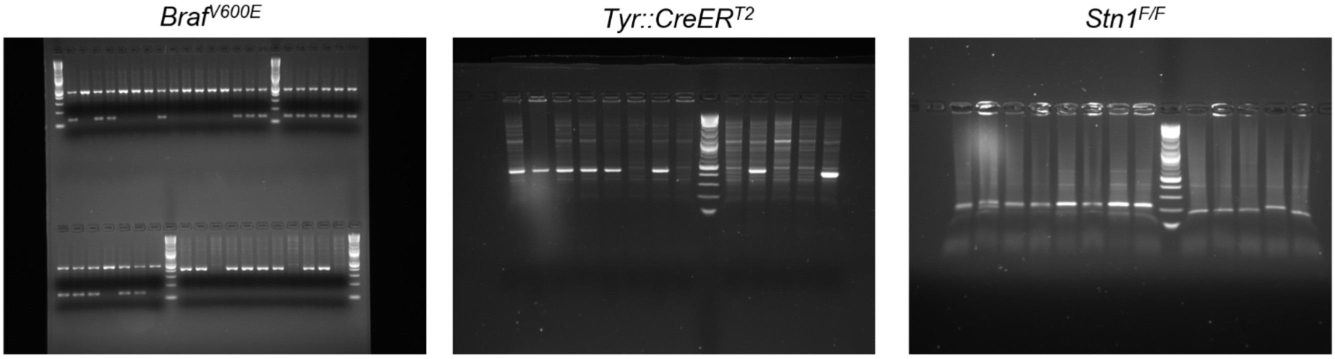

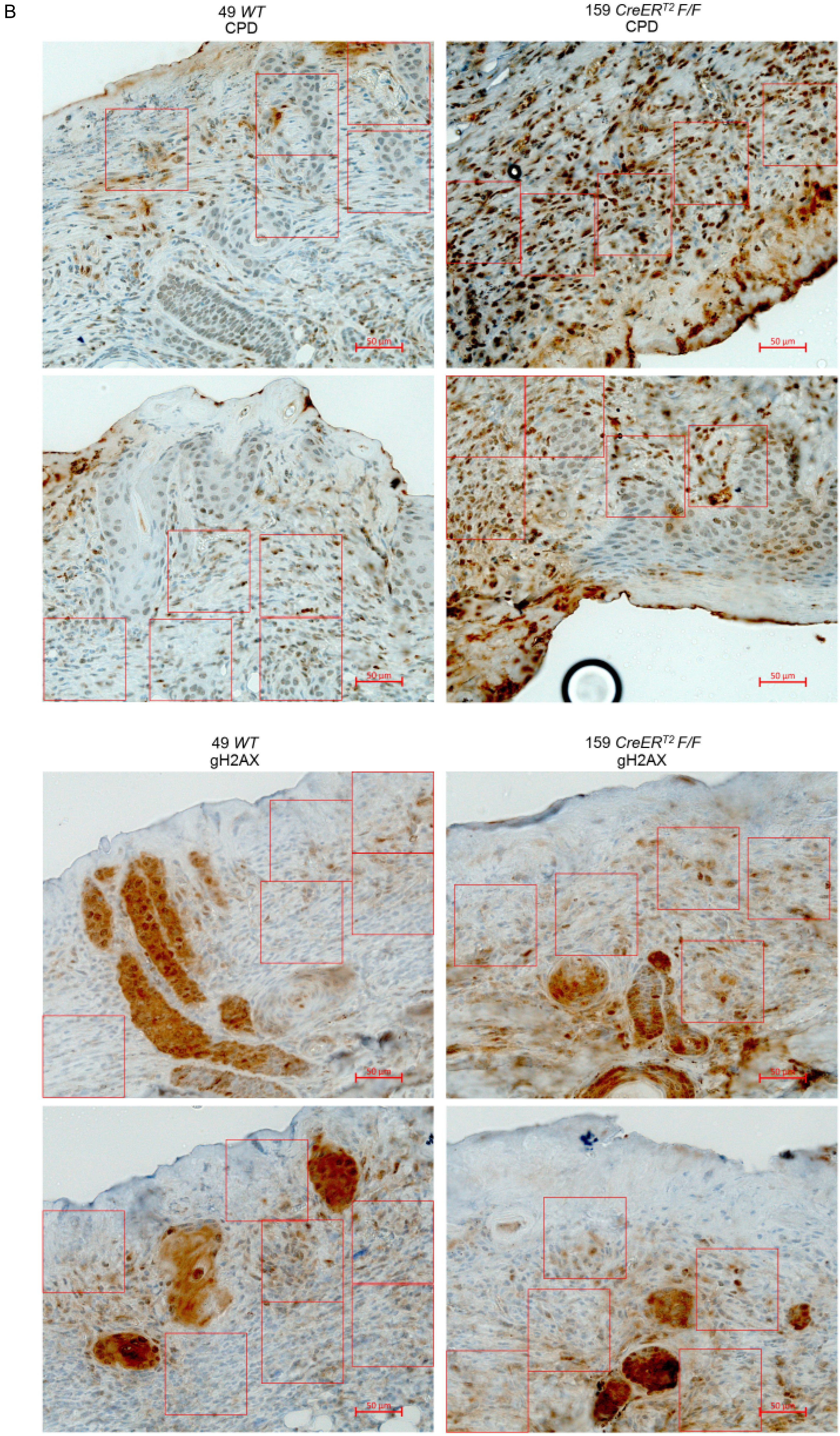
B
49 WT
CPD
159 CreER^T2 F/F
CPD
49 WT
gH2AX
159 CreER^T2 F/F
gH2AX
50 µm
