# Supplementary material for: In vivo investigation of STN1 downregulation in melanoma formation in adult mice following UV irradiation
Source: PLoS One. 2025 Nov 14;20(11):e0326647. doi: 10.1371/journal.pone.0326647 (PMC12617882; doi:10.1371/journal.pone.0326647)
Supplement: Attachment — Submitted filename: Comments_Pone.docx [file pone.0326647.s003.docx]

Comments

The authors have generated a Tamoxifen-inducible melanocyte specific Stn1 knock-out mouse model to study melanoma formation after UV exposure. They have used Braf-mutant animals as control for their experiments. The authors have previously shown that Stn1 deficiency makes mice susceptible to colorectal cancers, and the current study precisely shows that it might not be the case with melanoma. Although Stn1 is known to be downregulated in human melanoma patients, this might not be true in case of mouse models. This is a robust study and helps explain concerns in Stn1 mediated melanoma. I find this study suitable for publication after a few of my concerns are answered-

Major

All the previous in-vitro experiments have been performed in human cell lines. Based on these results the authors performed experiments in mouse models. It would be better if they could study the role of Stn1 in cell proliferation and DNA repair in primary melanocytes isolated from these animals under UV-exposure. The extent of DNA damage induced in melanocytes should be measured by gamma-H2AX or CPD levels. This may explain the observed phenotype.
