# Supplementary material for: In vivo investigation of STN1 downregulation in melanoma formation in adult mice following UV irradiation
Source: PLoS One. 2025 Nov 14;20(11):e0326647. doi: 10.1371/journal.pone.0326647 (PMC12617882; doi:10.1371/journal.pone.0326647)
Supplement: Attachment — Submitted filename: Rebuttal.docx [file pone.0326647.s004.docx]

Point-to-point response:

We are grateful for reviewers’ and editor’s constructive comments on our manuscript. We have performed additional experiments to address these comments. Our responses to the Editor’s and Reviewers’ comments are below in blue.

Additional Editor Comments:

This manuscript investigates whether STN1, a component of the CST complex important for genome stability, plays a role in UV-induced melanoma formation in vivo. The authors first show that CST genes are downregulated and sometimes altered in human melanoma datasets. They then generate a melanocyte-specific, tamoxifen-inducible STN1 conditional knockout mouse and chronically expose these mice to UV radiation. Contrary to expectations from in vitro studies, STN1 loss in mature melanocytes does not significantly alter melanoma incidence compared to controls. The work provides the first in vivo test of CST function in UV-driven melanoma but finds no clear effect under the experimental conditions. I have two major concerns.

1- IHC shows reduced Stn1 staining, but no functional assays (e.g., markers of replication stress, DNA damage) are provided to confirm that melanocytes lacking STN1 in vivo are indeed experiencing the expected genomic instability under UV stress. Such assays would strengthen the link between the negative tumor result and the molecular role of STN1.

Thank you for this point. We have performed additional experiments addressing this. We performed IHC staining of CPD and γH2AX on skin tissues from control and STN1 KO mice. Data are included in the new Figure 4, which shows that STN1 depletion in the mouse skin leads to increased UV lesions and DNA damage compared to control mice.

2- The knockout is induced in mature melanocytes. This leaves open the possibility that STN1 loss during melanocyte development, or in earlier life stages, might have different consequences. The restriction to adult deletion could mask effects relevant to melanoma initiation. Authors should acknowledge this limitation more explicitly.

Thank you for this point. We have pointed out this limitation of our study in Discussion in the initial submission (Page 17).

Reviewers' comments:

Reviewer's Responses to Questions

**Comments to the Author**

Reviewer #1: The paper requires more thorough statistical analysis on graphs (some is there). Additionally, the figures I was able to view were very low resolution making it more difficult to assess. I think they'v made an interesting observation, and I am interested in the gender differences they observe. I think it is worth speculating in one in two sentences based on literature potential examples of differential dna repair or influe4nces of hormones on replication stress and repair.

Thank you for pointing it out. The statistical p values were included in the main text in the original manuscript. We have now included the p values in relative plots.

We thank the reviewer for this insightful suggestion. Although the observed sex differences in our data did not reach statistical significance, we agree that hormonal influences may play a role in modulating DNA repair mechanisms. In the revised manuscript (Discussion, page ), we now include a speculative paragraph acknowledging that sex hormones have been reported to influence DNA damage response pathways. We also include the brief speculation that sex hormones can influence immunity that is important for regulating tumorigenesis. See Page 17-18.

Reviewer #2: Comments
The authors have generated a Tamoxifen-inducible melanocyte specific Stn1 knock-out mouse model to study melanoma formation after UV exposure. They have used Braf-mutant animals as control for their experiments. The authors have previously shown that Stn1 deficiency makes mice susceptible to colorectal cancers, and the current study precisely shows that it might not be the case with melanoma. Although Stn1 is known to be downregulated in human melanoma patients, this might not be true in case of mouse models. This is a robust study and helps explain concerns in Stn1 mediated melanoma. I find this study suitable for publication after a few of my concerns are answered-
Major

All the previous in-vitro experiments have been performed in human cell lines. Based on these results the authors performed experiments in mouse models. It would be better if they could study the role of Stn1 in cell proliferation and DNA repair in primary melanocytes isolated from these animals under UV-exposure. The extent of DNA damage induced in melanocytes should be measured by gamma-H2AX or CPD levels. This may explain the observed phenotype.

Thank you for this point. We have performed additional experiments addressing this. We performed IHC staining of CPD and γH2AX on skin tissues from control and STN1 KO mice. Data are included in the new Figure 4, which shows that STN1 depletion in the mouse skin leads to increased UV lesions and DNA damage compared to control mice.
